# No Differences Between 12 Weeks of Block- vs. Traditional-Periodized Training in Performance Adaptations in Trained Cyclists

**DOI:** 10.3389/fphys.2022.837634

**Published:** 2022-03-01

**Authors:** Nicki Winfield Almquist, Hanne Berg Eriksen, Malene Wilhelmsen, Håvard Hamarsland, Steven Ing, Stian Ellefsen, Øyvind Sandbakk, Bent R. Rønnestad, Knut Skovereng

**Affiliations:** ^1^Section for Health and Exercise Physiology, Inland Norway University of Applied Sciences, Lillehammer, Norway; ^2^Centre for Elite Sports Research, Department of Neuromedicine and Movement Science, Norwegian University of Science and Technology, Trondheim, Norway

**Keywords:** periodization, best practice, endurance training, skeletal muscle measures, hematological measures

## Abstract

The purpose of this study was to compare the effects of 12 weeks load-matched block periodization (BP, *n* = 14), using weekly concentration of high- (HIT), moderate- (MIT), and low- (LIT) intensity training, with traditional periodization (TP, *n* = 16) using a weekly, cyclic progressive increase in training load of HIT-, MIT-, and LIT-sessions in trained cyclists (peak oxygen uptake: 58 ± 8 ml·kg^−1^·min^−1^). Red blood cell volume increased 10 ± 16% (*p* = 0.029) more in BP compared to TP, while capillaries around type I fibers increased 20 ± 12% (*p* = 0.002) more in TP compared to BP from Pre to Post12. No other group differences were found in time-trial (TT) performances or muscular-, or hematological adaptations. However, both groups improved 5 and 40-min TT power by 9 ± 9% (*p* < 0.001) and 8 ± 9% (*p* < 0.001), maximal aerobic power (W_max_) and power output (PO) at 4 mmol·L^−1^ blood lactate (W_4mmol_), by 6 ± 7 (*p* = 0.001) and 10 ± 12% (*p* = 0.001), and gross efficiency (GE) in a semi-fatigued state by 0.5 ± 1.1%-points (*p* = 0.026). In contrast, GE in fresh state and VO_2peak_ were unaltered in both groups. The muscle protein content of β-hydroxyacyl (HAD) increased by 55 ± 58% in TP only, while both TP and BP increased the content of cytochrome c oxidase subunit IV (COXIV) by 72 ± 34%. Muscle enzyme activities of citrate synthase (CS) and phosphofructokinase (PFK) were unaltered. TP increased capillary-to-fiber ratio and capillary around fiber (CAF) type I by 36 ± 15% (*p* < 0.001) and 17 ± 8% (*p* = 0.025), respectively, while BP increased capillary density (CD) by 28 ± 24% (*p* = 0.048) from Pre to Post12. The present study shows no difference in performance between BP and “best practice”-TP of endurance training intensities using a cyclic, progressively increasing training load in trained cyclists. However, hematological and muscle capillary adaptations may differ.

## Introduction

Block periodization (BP) is a popular method for training organization used among coaches for elite athletes since the 1970’s (Issurin, 2010), and with scientific evidence for its effectiveness steadily accumulating. The main identifying factor of BP is a prioritized development of specific abilities in succession to avoid possibly conflicting stimuli by targeting specific stimuli in the blocked periods, thus contrasting traditional periodization (TP; Issurin, 2019).

A recent meta-analysis, summarizing the literature, demonstrated a small benefit of utilizing BP compared to TP of intervals on both maximal oxygen uptake (VO_2max_) and maximal aerobic power (W_max_; Molmen et al., 2019). Furthermore, this meta-analysis highlighted that the results diverged between studies, which may relate to small numbers of participants and generally low methodological quality. However, the divergence in results between studies applying a BP or TP might also reflect differences in content-, and distribution of training at different intensities (low-, moderate-, high-intensity training; LIT, MIT, and HIT, respectively), age, sex, and performance-level.

A cyclic, progressive increase in training load is a common feature of periodized training programs (Mujika et al., 2018), i.e., a progressive increase in duration, intensity, or the number of intervals. Even though this is viewed as a “best practice” approach, it is still a less stressed feature in studies comparing BT and TP. That being said, it has also been reported that it is “best practice” to use a polarized approach applying only LIT and HIT (Seiler, 2010). In line with the latter most BP vs. TP studies have focused on LIT and HIT, while MIT is almost excluded (Ronnestad et al., 2012, 2014, 2016; McGawley et al., 2017). Although HIT is shown to be superior to MIT only in improving VO_2max_ and W_max_ (Stoggl and Sperlich, 2014), it can be argued that MIT is an natural part of an well-designed endurance training schedule of endurance-trained individuals (Jones, 2006; Solli et al., 2019; van Erp et al., 2019). The inclusion of MIT could, arguably, be beneficial for improvements in performance at submaximal work rates.

On the molecular level, differentiating training stimuli might hypothetically help avoid stagnation in muscular adaptations (Goutianos, 2016). Indeed, mRNA-responses of the “master-switch” of mitochondrial adaptations, peroxisome proliferator-activated receptor γ co-activator-1α (PGC1α), have been reported gradually blunted (7-fold vs. 2-fold) when untrained subjects repeated the same endurance exercise session over time (Perry et al., 2010; Granata et al., 2019). Based on the above, it can be suggested that weekly changes in training focus and loads, induced by BP can induce greater molecular adaptations than the less weekly changes in a traditional evenly loaded TP. However, the molecular responses to BP and a TP with cyclic progressive increases in training load are thus far scarcely investigated (Goutianos, 2016).

However, whether the reported small beneficial effects of BP are mainly an effect of the change from a monotonous TP to a concentrated focus of BP or whether the results would be different by including MIT in both periodization approach is unclear. Hence, the effects of periodizing the major endurance intensity-modalities (i.e., HIT, MIT, and LIT) in a blocked periodization compared with a “best practice” TP, with cyclic, progressive loads using LIT, MIT, and HIT sessions has not yet been investigated. Also, the majority of studies finding no or only small differences between BP and TP in performance-related outcomes are of short duration (1–5 weeks; Breil et al., 2010; Clark et al., 2014; Ronnestad et al., 2016; McGawley et al., 2017), and it cannot be excluded that a longer duration might accentuate a possible difference in responses to BP and TP.

Therefore, the purpose of this study was to compare load-matched BP, using concentrated HIT-, MIT-, and LIT-weeks, with a TP using a mix of HIT-, MIT-, and LIT-sessions with a cyclic progressive increase in training load over 4 and 12 weeks in trained cyclists.

## Materials and Methods

### Participants

The current 12-week multi-center study involved two test-centers both completing a training intervention mid-winter (January–March) comparing BP with TP in a training load-matched parallel-group design. Participants were recruited from local cycling clubs near each test center. Fifteen cyclists participated at each test-centers, including four females and 26 males. All participants were categorized as trained with seven subjects at performance level 2, 14 participants at level 3, and seven at level 4–5 (De Pauw et al., 2013). Participants’ characteristics are shown in Table 1. The study was pre-registered with the Norwegian Social Science Data Services (NSD#: 61042), approved by the local ethical committee at Lillehammer University College, and performed according to the Declaration of Helsinki. All subjects were informed of the possible risks and discomforts associated with the study and provided their written informed consent before study participation.

**Table 1:** Participant’s characteristics and training volume.

	TP		BP		BP vs. TP
	Men (*n* = 14)	Women (*n* = 2)	Men (*n* = 12)	Women (*n* = 2)	
Age (Y)	35.5 ± 12.8	34.4 ± 4.9	40.5 ± 12.0	42.0 ± 6.6	*p* = 0.156
Body mass (kg)	83.9 ± 23.6	66.7 ± 8.1	79.9 ± 8.2	65.6 ± 0.2	*p* = 0.299
W_4mmol_ (W)	3.2 ± 0.6	2.6 ± 0.8	3.2 ± 0.6	2.6 ± 0.2	*p* = 0.878
VO_2peak_ (ml·kg^−1^·min^−1^)	59.4 ± 17.3	51.9 ± 5.7	57.1 ± 7.1	51.0 ± 5.5	*p* = 0.428
W_max_ (W·kg^−1^)	5.1 ± 1.5	3.9 ± 1.2	5.0 ± 0.8	4.1 ± 0.7	*p* = 0.836
40-min TT (W·kg^−1^)	3.3 ± 0.5	2.6 ± 0.2	3.4 ± 0.5	2.6 ± 0.4	*p* = 0.832
Training volume (hours·wk.^−1^)	6.7 ± 3.6	3.7 ± 3.0	6.9 ± 3.5	7.3 ± 1.4	*p* = 0.590

W_4mmol_ (W), power output at 4 mmol·L^−1^ blood lactate concentration; VO_2peak_, peak oxygen uptake; W_max_, maximal aerobic power output during the last minute of an incremental test to exhaustion; TT, time trial; and TV, training volume the month prior to inclusion in study (hours·wk.^−1^).

### Screening and Experimental Design

**Figure 1:**
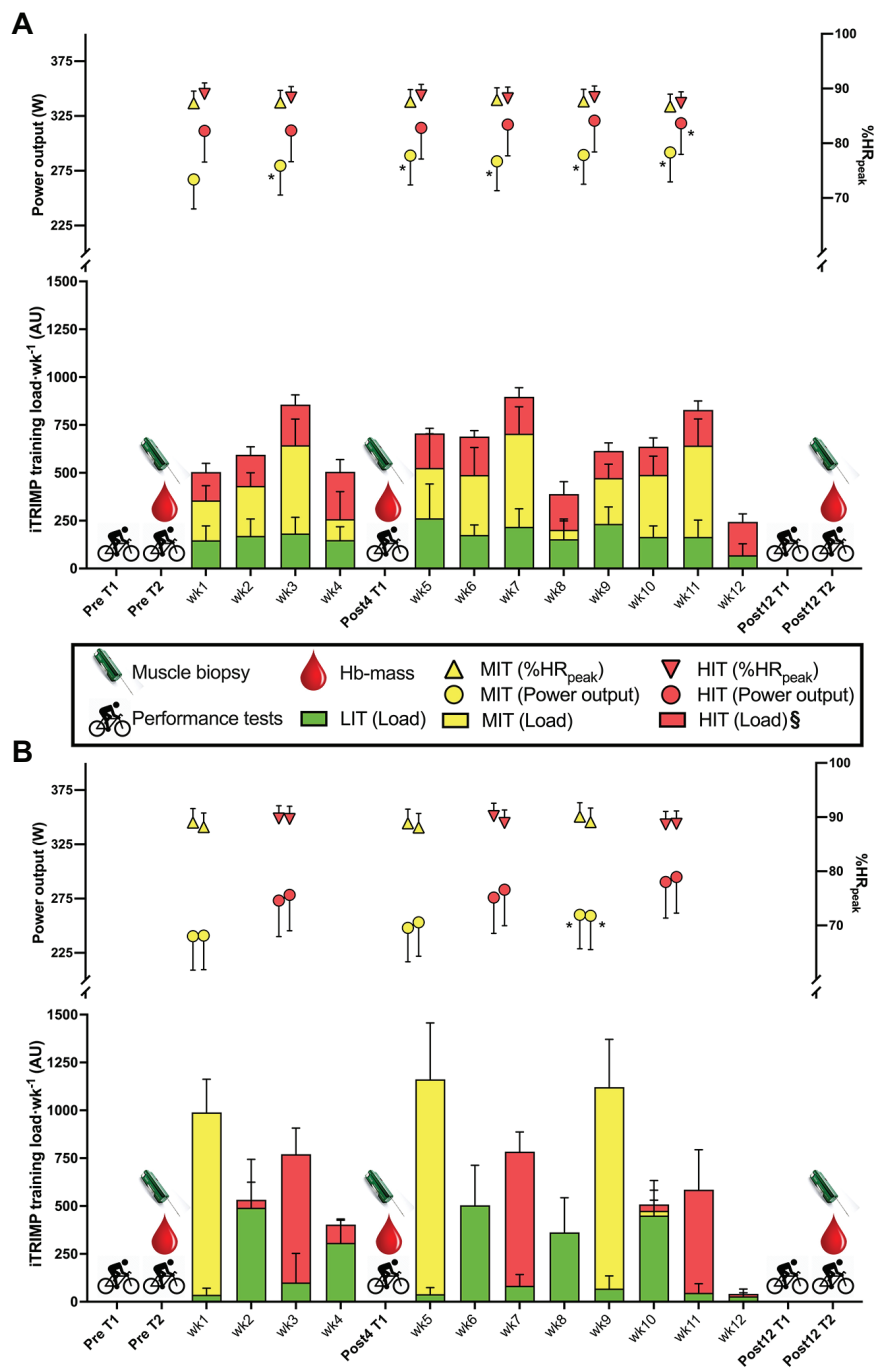
Experimental design and training load (iTRIMP) during a 4-week (Post4) and 12-week (Post12) intervention of either traditional periodization (TP, panel **A**) or blocked periodization (BP, panel **B**) of endurance training among 26 trained cyclists. Test day 1 (T1) included a blood lactate profile test, a peak oxygen uptake (VO_2peak_)-test and a 5-min time-trial (TT) performance test. Test day 2 (T2) included muscle biopsies, a 40-min TT and measurement of hemoglobin mass. Mean power output (PO) and % of peak heart rate (%HR_peak_) on the first and last moderate intensity training (MIT)- and high intensity training (HIT)-exercises of each mesocycle of 4 weeks are presented above the training load. Data are mean ± 95%CI. *Indicates a significant difference from the first MIT or HIT exercise (*p* < 0.05). § indicates a significant difference in HIT-load between TP and BP (*p* < 0.05). LIT, low-intensity training; MIT, moderate-intensity training; HIT, high-intensity training; Hb-mass, hemoglobin mass; and iTRIMP, individualized training impulse.

Before being included in the study, subjects performed a 40-min TT on their own bike, mounted on a bike trainer (Tacx Neo Smart, NL or Computrainer, Racermate, Seattle, United States). Heart rate (HR) and power output (PO) were recorded throughout, and blood lactate [BLa^−^] and rate of perceived exertion (RPE) were recorded every 5 min. Based on the average PO of a 40-min TT screening test and sex, the participants were pair-matched and randomly assigned to either BP or TP. After the physiological testing on test day 1 and 2 (see section “Testing procedures”) the participants completed three mesocycles of 4 weeks duration of either BP or TP (Figure 1). After the first meso-cycle a blood lactate profile test, a 6-s all-out sprint test, and a VO_2peak_ test were performed (Post4), while after the 12-week intervention, test days 1 and 2 were repeated (Post12).

### Intervention, Training Load, and Adherence

For the BP group, the 4-week mesocycle consisted of four MIT sessions in week 1, three LIT sessions in week 2, and four HIT sessions in week 3. For the TP group, the mesocycle consisted of alternating HIT, MIT, and LIT sessions evenly distributed throughout the first 3 weeks. The 4th week of each mesocycle was prescribed as a recovery week for both groups. Both groups were prescribed the same total number of HIT, MIT, and LIT sessions during each 4-week mesocycle and the HIT and MIT were made up of the same total number of efforts and had the same total duration. The recovery weeks were used as a catch-up for those who did not complete all MIT/HIT-sessions in the designated week. The Post12 test was performed on average 4 ± 2 days after the last MIT/HIT-session.

The participants were required to perform a minimum of four interval sessions (i.e., two MIT and two HIT) under the supervision of test leaders from the test centers, and they had the option for performing all HIT and MIT sessions under supervision. The supervised training was performed at the same location as all performance testing. Training was performed on personal bikes mounted on the same trainer throughout the study provided by the test centers. All LIT training was performed unsupervised, and other training forms such as XC-skiing and running were allowed only as LIT-sessions.

The HIT and MIT training were carried out as five efforts of 5 min and four efforts of 12 min, respectively, for the BP group. The TP group included a degree of progression in the sessions through each mesocycle and completed four, five, and six efforts for the three first HIT sessions and three, four, and five efforts for the three first MIT sessions. The last HIT and MIT session was identical between both groups to ensure that the last HIT stimulus was similar before recovery week and post-testing. All sessions were carried out using an effort-based approach. The participants were instructed to perform all sessions at the highest possible average power output without reducing the power output after the first effort. Target RPE scores for each session type with gradually increasing effort for each interval (i.e., MIT: 14–18 and HIT: 16–19) were provided as a guideline.

One male participant in the BP group dropped out due to the high training load. All participants completed the intervention but three male participants (two in the BP group and one in the TP group) were excluded from the final analysis due to insufficient compliance to the training intervention [i.e., too few completed sessions (<85%) or due to the completion of the block periods over too many days (>6)].

#### Training Load Quantification

The training was registered in a commercially available online training diary (trainingpeaks.com, Colorado, United States). The participants were required to register all training (i.e., HIT, MIT, LIT, and strength training and activity mode) during the entire intervention period. Training load was quantified using the iTRIMP as described elsewhere (Manzi et al., 2009), by weighting exercise intensity according to an individual’s own HR vs. [Bla^−^] relationship, calculated by the line of best fit from the lactate profile and VO_2peak_ test. iTRIMP uses the weighting factor y_i_, which increases exponentially based on the HR vs. [Bla^−^] relationship to weight every HR. An accumulated iTRIMP score was calculated by the following equation:


$$\mathrm{iTRIMP}\;\left[\mathrm{arbitrary units}\;\left(\mathrm{AU}\right)\right]=D\;\left(\min\right)\times \Delta HR_{\mathrm{ratio}}\times yi$$

where Δ*HR*_ratio_ is calculated from (HR_work_-HR_rest_)/(HR_max_-HR_rest_), and *D* is time spent exercising.

#### Training

The average adherence to the training for the participants included in the final analysis was 92 ± 8%. Data from 12 (i.e., six HIT and six MIT) of the supervised sessions showed an expected effect of session design, with greater power output during the HIT sessions compared to the MIT sessions (*p* < 0.01), also reflected by a higher [Bla^−^] (*p* < 0.01). However, the average session RPE was not different between the supervised HIT and MIT sessions (*p* = 0.760).

The average weekly training volume (hrs·wk.^−1^) during the intervention was not different between groups (*p* = 0.571) with an average of 7.5 ± 2.0 h·wk.^−1^ in TP and 8.0 ± 2.7 h·wk.^−1^ in BP, respectively. Hence, the increase in training volume from habitual training was not different between groups (*p* = 0.938) with an increase of 43 ± 89% in TP and 46 ± 125% in BP.

The training load (i.e., iTRIMP scores) for the whole intervention period was not different between the groups (*p* = 0.820) and there was no difference in the average iTRIMP score from the LIT and MIT sessions (both *p* > 0.54; Figure 1). The BP group had a higher iTRIMP score in the HIT sessions (*p* > 0.05), which was reflected by a higher session-RPE compared to the TP group (*p* < 0.05), whereas no difference in session-RPE was observed for MIT sessions (*p* = 0.07). Time spent in the five heart rate zones, as defined by Seiler (2010), over the whole study was not different between BP and TP (all *p* > 0.09), and there was no difference between BP and TP in [Bla^−^] (*p* = 0.47).

Power output during MIT and HIT sessions increased from the beginning to the end of the intervention period in both groups (*p* < 0.05) and both the increase during the intervention period (*p* = 0.64) and the difference in power output between the MIT and HIT sessions (*p* = 0.96) did not differ between the groups. Across groups there was a tendency for [Bla^−^] to decrease during the intervention period (*p* = 0.06), whereas RPE (*p* = 0.29) did not change.

### Testing Procedures

The day prior to each experimental day of testing, the participants were instructed to perform a standardized, self-organized 1-h LIT-session.

#### Test Day 1 (T1): Physiological Tests

##### Blood Lactate Profile Test

The blood lactate profile test has previously been described (Almquist et al., 2020) and consisted of 5-min stages with incremental load (50 W). When the participants reached a blood lactate of 3 mmol·L^−1^, the load increases were reduced to 25 W. The blood lactate profile was terminated when blood lactate exceeded 4 mmol·L^−1^. VO_2_ measurements started from 2 min into every bout and VO_2_ was calculated as an average from 2.5 to 4.5 min and blood was sampled from the fingertip on completion of each 5-min bout and analyzed for whole blood [La^−^] using a lactate analyzer (Biosen C line, EKF Diagnostic, Germany). HR was recorded using the participants’ own HR-monitor and RPE was recorded according to Borg Scale 6–20. VO_2_ was measured using a computerized metabolic system with a mixing chamber (Oxycon Pro, Erich Jaeger, Hoechberg, Germany), which was calibrated every hour and all cycling was performed on an electromagnetic braked cycle ergometer (Lode Excalibur Sport, Lode B. V., Groningen, The Netherlands) which was adjusted to each cyclists’ individual preferences and replicated throughout all testing.

##### 6-s All-Out Sprint

After a 5-min active recovery a 6-s all-out sprint test was performed in the seated position using the *Wingate* modus with a stationary start and a resistance of 0.8 Nm·kg^−1^ body mass. Peak power output was defined as the highest value achieved during the 6-s all-out, with recordings at 5 Hz.

##### VO_2peak_ Test

Following another 5-min active recovery, a VO_2peak_ test was performed. The test was performed using an incremental ramp protocol with 25 W increments each minute until voluntary exhaustion or the inability to maintain a cadence >60 rpm. The starting work rate was individually adjusted based on the lactate profile test to ensure exhaustion in 8–12 min. VO_2peak_ was calculated as the highest average of a 1-min moving average using 5-s VO_2_-measurements and peak heart rate (HR_peak_) was registered. W_max_ was calculated as the mean power output during the last minute of the incremental test.

##### 45 min Continuous Cycling and 5-min TT

After 10 min of active recovery the participants cycled for 30 min at an intensity corresponding to 60% of VO_2peak_ after which the third-last and second-last stages from the blood lactate profile test were repeated followed by another 5 min at 60% of VO_2peak_. This was immediately followed by a 5-min self-paced TT, where the participants were instructed to achieve the highest possible mean power output. The start power output was replicated at Post to ensure the same pacing conditions. VO_2_, HR, RPE, and [Bla^−^] were measured during the repeated stages from the blood lactate profile test and during the 5-min TT. VO_2_-measurements started 30-s prior to each period to ensure steady measures of VO_2_. Water, energy-drink (HIGH-5, United Kingdom), and gels (SIS Isotonic Energy Gel, United Kingdom) without caffeine were provided *ad libitum* after the incremental test to exhaustion and throughout the test. The amount was recorded and repeated at Post12 to ensure the same relative hydration level. Gross efficiency (GE) was defined as the ratio between the mechanical PO and the metabolic power input (PI) calculated using VO_2_ measurements and the energetic equivalent (Peronnet and Massicotte, 1991) PI = VO_2_ L·s^−1^ × (4,840 J·L^−1^ × RER + 16,890 J·L^−1^). GE was calculated from the blood lactate profile test in the fresh state and from the two repeated stages in the fatiguing state during 45 min continuous cycling.

#### Test Day 2 (T2): Muscle Sampling and 40-min TT

The participants arrived at the laboratory at least 2 h after the last, standardized meal and rested for 20 min in a supine position. Two muscle biopsies were collected, using the micro-biopsy technique as described elsewhere (Hayot et al., 2005) with a 14-gauge needle (Bard Magnum, Bard Nordic, Helsingør, Denmark) in the resting state under local anesthesia (Xylocaine, 10 mg·ml^−1^, AstraZenaca AS, Oslo, Norway) from the m. Vastus Lateralis of a randomized leg. Biopsies were sampled from the same leg Pre, Post4, and Post12, approximately 2 cm proximal to the previous sample. One muscle sample was immediately snap-frozen in isopentane (−80°C) and stored at −80°C until further analyses of muscle protein content and activity, while the second biopsy was quickly dissected free of blood and visible connective tissue in ice-cold sterile saline solution (0.9% NaCl) and transferred to a 4% formalin solution for fixation for 24–72 h, before further preparation for immunohistochemistry.

##### 40-min TT

A standardized 16-min warm-up was performed prior to a 40-min TT. The participants performed the 40-min TT on their own bikes mounted on the same bike trainer (Tacx Neo Smart, Wassenaar, NL or Computrainer, Racermate, Seattle, United States). HR and power output were recorded throughout and [Bla^−^] and RPE were recorded every 5 min. The participants were allowed to see the instantaneous power output but were blinded to the average power throughout the test. The participants were instructed to obtain the highest average power output during the test and encouraged to stay seated but standing cycling was allowed. After completion of the test, mean power output, mean and peak HR, RPE, and [Bla^−^] were registered.

##### Hematological Measures

Hematological measures were performed on a subset of the participants (*n* = 8 for TP, and *n* = 6 for BP) due to lack of equipment at one test center. The participants rested for 20 min in a semi-recumbent position and Hb-mass was determined using a modified version of the carbon monoxide (CO) rebreathing technique, as described elsewhere (Siebenmann et al., 2012). Hb-mass was determined using OpCO (Detalo Performance, Detalo Health, Birkerød, Denmark). Briefly, the participant breathed 100% O_2_ for 3 min before a blood sample was drawn from the antecubital vein (125 ml) using pre-heparinized syringes (PICO50 80IU, Radiometer, DK) and immediately analyzed in triplicate for carboxy-Hb (%HbCO) on a hemoximeter (ABL800, Radiometer, Copenhagen, Denmark). Subsequently, the participants rebreathed a bolus of chemically pure CO (Multigas SA, Domdidier, Switzerland) corresponding to 1.5 ml·kg^−1^ for men and 1.0 ml·kg^−1^ for women for 9 min. A sensor registered and regulated the O_2_-level during the rebreathing. After rebreathing, another blood sample was drawn from the antecubital vein, analyzed for %HbCO in triplicate. The change in %HbCO between first and second measurement was used to calculate Hb-mass. Total red blood cell volume (RCV), total blood volume, and plasma volume was calculated from Hb-mass and Hb-concentration with the following calculations as described earlier (Siebenmann et al., 2015):


Hbmass=644×nCOabs×25/ΔHbCO

where *∆HbCO* is the change in *%HbCO* between the blood sample before and after administration of CO-dose.


$$\mathrm{RBCV}\;\left(\mathrm{ml}\right)=\mathrm{Hbmass}\times Hct/\left[Hb\right]$$

$$BV\;\left(\mathrm{ml}\right)=\mathrm{RBCV}\times 100/Hct$$

$$PV\;\left(\mathrm{ml}\right)=BV–\mathrm{RBCV}$$

### Muscle Analyses

#### Western Blotting

Preparation and analyses of muscle tissue were conducted using the same protocol as previously described (26). Samples were homogenized for ~120 s using a plastic pestle in 80 ml·mg^−1^ fresh lysis buffer [2 mM HEPES, pH 7.4; 1 mM EDTA, pH 7.0; 5 mM EGTA, pH 7.5; 10 mM MgCl_2_; 1% Triton-X-100; phosphatase, and protease inhibitors]. Subsequently to homogenization the samples were rotated end-over-end for 1 h and centrifuged for 10 min at 10,000 *g* to separate undissolved tissue from the supernatant. Afterward, the supernatant was carefully separated from the pellet and stored at −80°C until further analysis. Protein concentration was determined using the Pierce Detergent Compatible Bradfor Assay Kit #23246. Briefly, 5 ml samples were diluted 1:10 in ddH_2_O and loaded in triplicates onto a 96-well microtiter plate, mixed with 250 ml Pierce Detergent Compatible Bradford Assay Reagent, and measured spectrophotometrically at 595 nm using a Multiscan FC microplate reader (Thermo Fisher Scientific), using the SkanIt software 2.5.1 for Multiscan (Thermo Scientific). Pierce Serum Albumin standards with protein concentrations ranging from 0.025 to 2.0 mg·ml^−1^ were used to create a standard curve. Protein concentrations were calculated from the standard curve after correction for the absorbance of the ddH_2_O.

The lysates were normalized to a protein concentration of 2.0 mg·ml^−1^ in ice-cold fresh buffer containing: 10% glycerol, 20 mM Na-pyrophosphate, 150 NaCl, 50 mM HEPES (pH 7.5), 1% NP-40, 20 mM β-glycerophosphate, 2 mM Na3VO4, 10 mM NaF, 2 mM PMSF, 1 mM EDTA (pH 8), 1 EGTA (pH 8), 10 μg/ml Aprotinin, 10 μg/ml Leupeptin, and 3 mM Benzamidine. The samples were subsequently rotated end-over-end for 1 h at 4°C and centrifuged at 18,320 *g* for 20 min at 4°C to exclude non-dissolved structures. The lysates were prepared with a 4x Laemmli sample buffer (Bio-Rad Laboratories AB, Oslo, Norway) containing 10% 2-Mercaptoethanol and heated for 5 min at 95°C. Proteins samples (15 mg of total protein) were separated at 300 V for 60 min using an Invitrogen gel (4–20% Criterion™ TGX™ Precast Midi Protein Gel, 26 well, 15 μl), followed by wet transfer to a PVDF membrane (0.2 mm Immuno-Blot, Bio-Rad) at 400 mA for 60 min. For each participant, all samples were loaded on the same gel in technical duplicates. Membranes were then stained using a reversible total protein stain (Pierce Reversible Protein Stain, Thermo Fischer Scientific) to ensure appropriate protein transfer and to control for loading. Membranes were then blocked using 3% Bovine Serum Albumin in Tris-buffered saline including 0.1% Tween-20 (TBST) for 60 min at room temperature, before overnight incubation in primary antibody on a rocking table at 4°C. Membranes were then washed 2 × 5 min in TBST, followed by incubation in a TBST-solution containing 5% skimmed milk and horseradish-peroxidase-conjugated secondary antibody for 60 min at room temperature. The membranes were then washed 4 × 5 min in TBS-T, and bands were visualized using chemiluminescent detection (Immobilon Forte, Western HRP Substrate, Millipore) and recorded with a digital camera (ChemiDoc XRS+, BioRad Laboratories). Band intensities were quantified using Image Lab 6.0.1 (Bio-Rad, Laboratories), adjusted for background intensity. Samples were normalized to a human pool (HP) containing equal amounts of all Pre-samples, which was loaded onto each gel in duplicates. Primary antibodies were purchased from Abcam; Anti-Citrate synthase (CS), 1:2,000 (ab96600), anti-cytochrome c oxidase subunit IV (COXIV), 1:4,000 (ab156056), anti-β-hydroxyacyl (HAD), 1:8,000 (ab154088), Santa Cruz Biotechnology; phosphofructokinase 1 (PFK-1), 1:500 (sc166722), and Thermo Fischer Scientific; Sodium-potassium pump β1 subunit (Na^+^-K^+^β1), 1:1,000 (MA3-930), and DSHB; Sodium-potassium pump α subunits (Na^+^-K^+^α), 1:60 (alfa5-S).

#### Enzyme Activity

Citrate synthase and phosphofructokinase (PFK) activity were assayed in muscle lysates using commercially available kits (CS: CS0720, PFK: MAK093, St. Louis, MO, Sigma-Aldrich) according to the manufacturer’s instructions as described previously (42). All activities were normalized to protein concentration as described above and expressed in international mU·mg^−1^ protein.

#### Histochemical Analyses of Muscle Fiber Size and Capillarization

Formalin-fixed muscle biopsies were processed rapidly using a Shandon Excelsior ES (Thermo Fisher Scientific, Waltham, MA, United States). After that, biopsies were paraffin-embedded and sectioned into 4 μm transverse sections. Antigen retrieval was performed at 97°C for 20 min in a target retrieval solution (cat. no. DM828, Agilent Dako, Santa Clara, CA, United States) using a PT link (PT 200, Agilent Dako, Santa Clara, CA, United States). Staining was performed using a DAKO Autostainer Link 48 (Agilent Dako, Santa Clara, CA, United States). To determine muscle fiber cross-sectional area, fiber type, numbers of myonuclei per muscle fiber type, capillary density (CD), capillaries around fibers (CAF) per fiber type, and capillaries to fiber ratio (*CF*), cross-sections were triple stained using primary antibodies against muscle fiber membrane (dystrophin, diluted 1:100, cat. no. PA1-37587; Thermo Fisher Scientific, Waltham, MA, United States), myosin heavy chain I (diluted 1:2,000, cat. no. M8421, Sigma-Aldrich, Saint-Louis, MO, United States), and CD34 (diluted 1:50, cat. no. M7165, DAKO Agilent). Visualization was achieved using the secondary antibodies Alexa Fluor 594 (IgG H + L, diluted 1:400, cat. no. A11037) and 488 (IgG1γ1, diluted 1:400, cat. no. A21121), respectively (Thermo Fisher Scientific, Waltham, MA, United States). CD34 was visualized using EnVision™ high pH (Link) kit (cat. no K8000, DAKO Agilent). Muscle sections were then covered with a coverslip and glued with EverBrite™ Hardset Mounting Medium containing DAPI (cat. no. 23004, Biotium Inc., Fremont, CA, United States) to visualize cell nuclei. Images of stained cross-sections were captured using a high-resolution camera (Axiocam, Zeiss, Oberkochen, Germany) mounted on a light microscope (Axioskop-2, Zeiss, Oberkochen, Germany), with a fluorescent light source (X-Cite 120, EXFO Photonic Solutions Inc., Mississauga, Canada). Multiple images were taken using 10× objectives to capture the entirety of each cross-section. Analyses were performed using an automated procedure CellProfiler 4.2.1 (Carpenter et al., 2006) ensuring an unbiased quantification. On average, 234 ± 155 fibers were analyzed per muscle sample.

### Statistics

All variables were tested for normal distribution using the Shapiro–Wilk test and were log-transformed to obtain normality if not normal. To compare relative changes in physiological, performance, muscular (% changes from Pre), and hematological measures from Pre to Post4 and Pre to Post12 between groups, a mixed linear model was applied with group defined as fixed effects and corrected using Pre-values as a covariate using the software SPSS v.25. For all immunohistochemical measures (CSA, fiber type proportion, capillary, and nuclei measures), the models were weighted for the number of fibers counted in each sample to account for the reduced reliability of fewer fibers. To compare main effects of time and group a mixed linear model was applied with fixed effects defined by group and time and random effects were defined by subject. Performance and physiological measures are presented as mean ± SD, while estimated marginal means (EMM) of % changes in muscular measures are mean ± 95%CI. *Post hoc* analyses were performed with Sidak adjustments using an alpha-level of 0.05.

## Results

### Performance Measures

**Figure 2:**
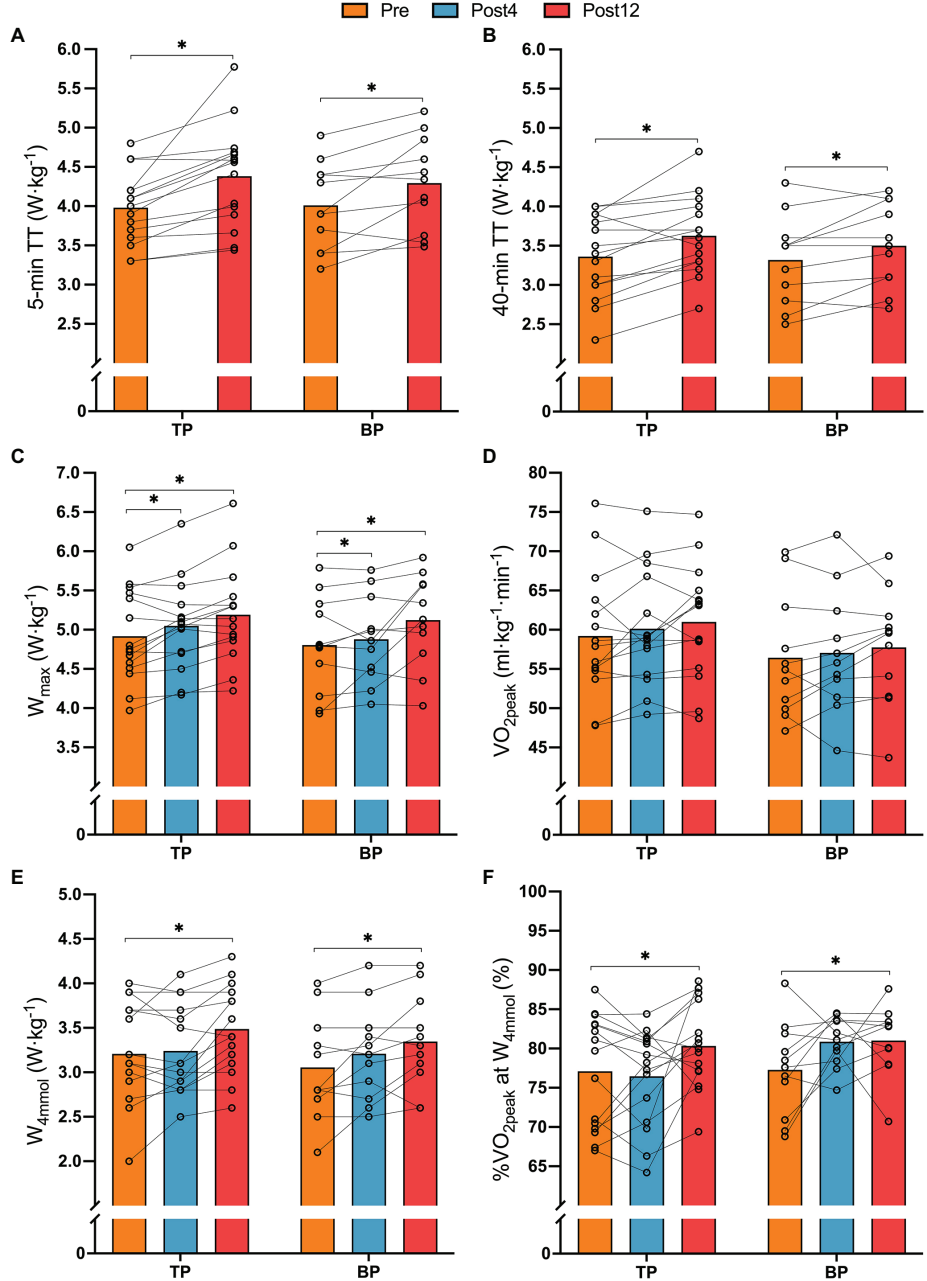
Relative mean PO on 5-min TT (panel **A**), relative mean power output on 40-min TT (panel **B**), relative maximal aerobic power output (W_max_, panel **C**), relative peak oxygen uptake (VO_2max_, panel **D**), relative power output at 4 mmol·L^−1^ [BLa^−^] W·kg^−1^ (panel **E**), and fractional utilization of VO_2peak_ at 4 mmol·L^−1^ [BLa^−^] (panel **F**) from before (Pre) to after 4- (Post4) and 12 weeks (Post12) of TP (*n* = 15) or BP (*n* = 11). *Indicates a main effect of time (*p* < 0.05).

There were no differences in the effect of training between TP and BP in 5-min (*p* = 0.940) and 40-min TT (*p* = 0.612) power output relative to body mass, but when pooling the groups, training increased 5 and 40-min TT power output by 8.9 ± 8.9% (*p* < 0.001) and 8.4 ± 9.0% (*p* < 0.001), respectively (Figures 2A–C). Likewise, for W_max_, there was no difference in the effect of training between TP and BP (*p* = 0.511), but pooled data showed a 2.4 ± 4.5% (*p* = 0.016), and a 6.3 ± 6.6% (*p* = 0.001) improvement of W_max_, respectively, from Pre to Post4 and Pre to Post12.

### Performance-Related Measures

There was no difference between TP and BP in the effect of training on VO_2peak_ (interaction effect: *p* = 0.94), and neither group changed VO_2peak_ from Pre to Post4 or Post12 (all *p* > 0.11; Figure 2D). There was no difference between TP and BP in the effect of training on W_4mmol_ (interaction effect: *p* = 0.31, Figure 2E) or on the fractional utilization of VO_2_ at W_4mmol_ (interaction effect: *p* = 0.100; Figure 2F). However, pooled data showed an effect of time where W_4mmol·kg_^−1^ increased by 10.2 ± 12.4% (*p* = 0.001) from Pre to Post12 and the fractional utilization of VO_2_ at W_4mmol_ increased in by 5.0 ± 8.5%-points (*p* = 0.026).

Likewise, there was no difference between TP and BP in the effect of training on maintaining GE from the fresh to the semi-fatigued state (interaction effect: *p* = 0.34). Pooled data showed that training reduced the deterioration of GE by 0.5 ± 1.1%-points (*p* = 0.026) from Pre to Post12. %VO_2peak_ during the 5-min TT, %HR_peak_ during the 40-min TT, and 6-s all-out peak power was not affected by training in BP or TP (Table 2).

**Table 2:** Body mass, performance-related measures, and 6-s all-out sprint peak power before (Pre) to after 4 (Post4) and 12 weeks (Post12) of traditional periodization (TP, *n* = 15) and blocked periodization (BP, *n* = 11).

	TP		BP	
	Pre	Post12	Pre	Post12
Body mass (kg)	82.3 ± 11.2	81.6 ± 9.8	76.5 ± 9.2	76.4 ± 8.8
%VO_2peak_ 5-min TT (%)	88.5 ± 4.4	90.0 ± 4.3	89.4 ± 5.8	90.2 ± 4.1
%HR_peak_ 40-min TT (%)	91.6 ± 4.2	91.3 ± 3.7	93.1 ± 2.4	92.3 ± 3.8
GE fresh (%)	19.4 ± 1.9	19.2 ± 1.6	18.5 ± 2.2	18.7 ± 2.3
GE semi-fatigued (%)	18.5 ± 2.1	18.8 ± 1.6^*^	18.0 ± 1.4	18.6 ± 2.0^*^
GE fresh vs. semi-fatigued	−0.9 ± 0.9	−0.3 ± 0.8^*^	−0.5 ± 1.1	−0.1 ± 1.0^*^
6-s peak power (W·kg^−1^)	17.0 ± 3.3	17.1 ± 3.1	15.0 ± 3.7	15.3 ± 3.6

[BLa^−^], blood lactate concentration measured 1 min after conclusion of 5 and 40-min time-trial (TT); RPE, rate of perceived exertion immediately after 5 and 40-min TT; GE, gross efficiency measured in steady-state periods in the fresh and the semi-fatigued state during the ~2-h long test protocol; and 𝞓GE, change in gross efficiency from the fresh to the semi-fatigued state (%- points).

* Indicates a significant change from Pre to Post12 (*p* < 0.05).

### Skeletal Muscle Measures

**Figure 3:**
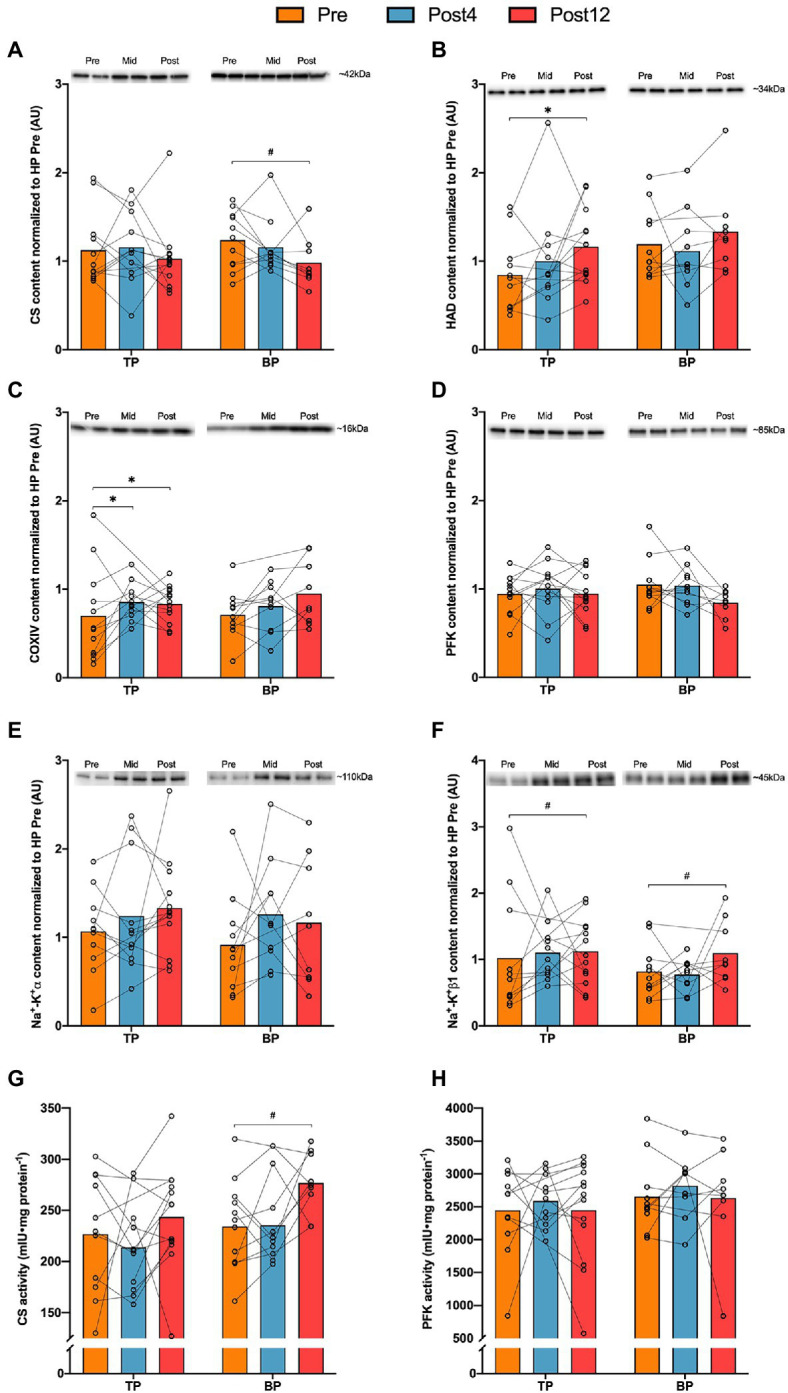
Muscle protein quantities (panels **A–D**) and activities (panels **E,F**) in m. vastus lateralis before and after 4 (Post4) and 12 weeks (Post12) of TP (*n* = 13) and BP (*n* = 10). Panel **(A)**, Citrate synthase (CS); panel **(B)**, β-hydroxyacyl (HAD); panel **(C)**, Cytochrome c oxidase subunit 4 (COXIV); panel **(D)**, phosphofructokinase (PFK); panel **(E)**, Sodium-potassium pump α (Na^+^-K^+^α); panel **(F)**, Sodium-potassium pump β1 (Na^+^-K^+^β1); panel **(G)**, enzyme activity of citrate synthase; and panel **(H)**, enzyme activity of phosphofructokinase. Individual band-intensities were expressed relative to total protein stain and normalized to a human pool (HP) containing equal amounts of all Pre-samples. Mean and individual values. *Indicates *post hoc* effect of time (*p* < 0.05).

There was no difference between TP and BP in any protein content alteration from Pre to Post4 or Pre to Post12 (Figure 3). Pooled data showed an effect of time for HAD (*p* = 0.037), COXIV (*p* = 0.005), Na^+^-K^+^α (*p* = 0.036), and Na^+^-K^+^β1 (*p* = 0.015). *Post hoc* analyses revealed a 55 ± 58% (*p* = 0.048) increase in HAD protein content in TP from Pre to Post12, while protein content of COXIV increased in both TP and BP from Pre to Post12 by 88 ± 54% (*p* = 0.037), and 79 ± 60% (*p* = 0.041), respectively. Enzyme activity of CS and PFK did not change differently between groups. However, for CS there was an effect of time (*p* = 0.043) when pooling the groups, but *post hoc* tests neither showed alterations in TP (12 ± 35%, *p* = 0.367) nor BP (22 ± 33%, *p* = 0.051) from Pre to Post12. No other changes were observed within either group from Pre to Post4 or Post12.

### Muscle Morphological and Capillary Measures

Traditional periodization led to different alterations in C/F-ratio (interaction *p* = 0.045) and CAF type I (interaction *p* = 0.049) than BP. C/F-ratio increased 19 ± 18% (*p* = 0.038) more in TP from Pre to Post4 compared to BP, and CAF type I increased 20 ± 12% (*p* = 0.002) more in TP from Pre to Post12 than BP (Table 3). The effect of training was otherwise similar between groups.

Generally, for pooled data, there was an effect of time for CD (*p* = 0.011), C/F-ratio (*p* < 0.001), CSA type II fibers (*p* = 0.032), CAF type II fibers (*p* = 0.024), and nuclei per fiber type II (*p* = 0.042). Specifically in TP, C/F-ratio increased by 27 ± 12% (*p* = 0.021) and by 36 ± 15% (*p* < 0.001) from Pre to Post4 and Post12, respectively, while CAF type I increased by 17 ± 8% (*p* = 0.025) from Pre to Post12. In BP, CSA of type II fibers decreased by 19 ± 11% (*p* = 0.032) from Pre to Post4, while CD increased by 28 ± 24% (*p* = 0.048) in BP from Pre to Post12. Nuclei per fiber type I were lower in BP compared to TP at Post4 (*post hoc p* = 0.010). *Post hoc* did not show any other changes within either group from Pre to Post4 or Post12.

### Hematological Measures

**Figure 4:**
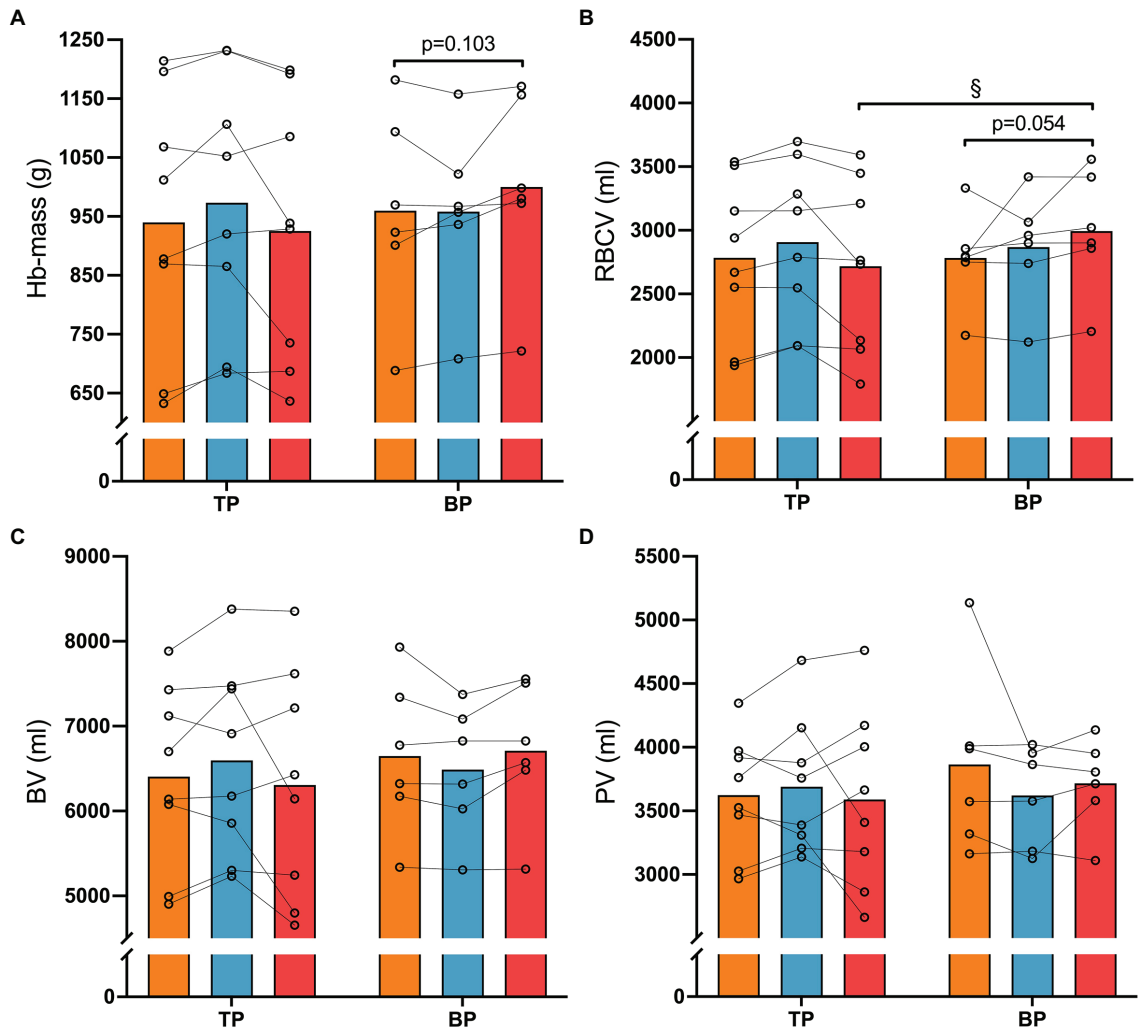
Haematological measures before (Pre), after 4 weeks (Post4), and after 12 weeks (Post12) of traditional periodization (TP, *n* = 8) and blocked periodization (BP, *n* = 6). Panel **(A)**, Haemoglobin mass (Hb-mass); Panel **(B)**, Red blood cell volume (RBCV); Panel **(C)**, Blood volume (BV); Panel **(D)**, Plasma volume (PV). § indicates an interaction (time × group, *p* < 0.05).

There was a different effect of training between TP and BP on RBCV (interaction effect *p* = 0.017) and HB-mass (interaction effect *p* = 0.014; Figure 4) in favor of BP. Specifically, BP increased RBCV 10 ± 16% (*p* = 0.029) more than TP from Pre to Post12 (TP: −3 ± 7% vs. BP: 7 ± 8%), while *post hoc* analyses showed no differences in HB-mass changes between TP and BP (TP: −1 ± 7% vs. BP: 5 ± 4%, *p* = 0.103) from Pre to Post12. No other differences were observed between or within TP and BP from Pre to Post4 or Post12.

**Table 3:** Skeletal muscle morphological measures, capillarization and cell nuclei from before (Pre) to after 4 weeks (Post4), and 12 weeks (Post12) of TP (*n* = 13) and BP (*n* = 11).

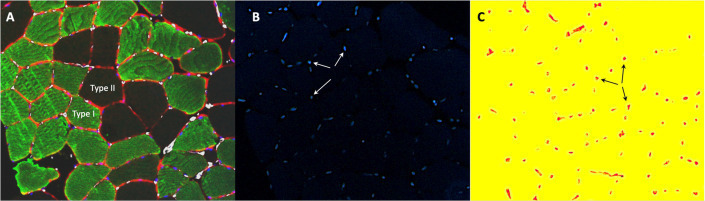						
	TP			BP		
	Pre	Post4	Post12	Pre	Post4	Post12
Type I fibers (%)	66 ± 7	68 ± 6	69 ± 5	67 ± 6	69 ± 7	62 ± 6
CD (cap/mm^2^)	418 ± 79	463 ± 74	494 ± 63	426 ± 72	505 ± 83	518 ± 71^*^
C/F-ratio (cap/fiber)	1.7 ± 0.3	2.2 ± 0.3^*^^§^	2.4 ± 0.3^*^	1.8 ± 0.3	1.8 ± 0.4	2.1 ± 0.3
CSA type I fibers (μm^2^)	4,379 ± 597	4,533 ± 569	4,803 ± 531	4,226 ± 581	3,787 ± 637	4,231 ± 584
CSA type II fibers (μm^2^)	4,537 ± 802	4,816 ± 786	5,041 ± 744	4,802 ± 797	3,897 ± 865^*^	4,892 ± 791
CAF type I	5.5 ± 0.9	5.5 ± 0.9	6.7 ± 0.8^*^^§^	5.1 ± 0.9	4.9 ± 1.0	4.9 ± 0.9
CAF type II	5.1 ± 1.0	4.8 ± 0.9	5.8 ± 0.8	4.8 ± 0.9	3.9 ± 1.0	4.6 ± 0.9
CAFA type I (CAF/CSA)	1.3 ± 0.3	1.2 ± 0.2	1.4 ± 0.2	1.3 ± 0.2	1.4 ± 0.3	1.3 ± 0.2
CAFA type II (CAF/CSA)	1.2 ± 0.2	1.0 ± 0.2	1.2 ± 0.2	1.0 ± 0.2	1.2 ± 0.3	1.1 ± 0.2
Nuclei per fiber type I	2.3 ± 0.4	2.7 ± 0.3^#^	2.7 ± 0.3	2.2 ± 0.3	2.0 ± 0.4	2.4 ± 0.3
Nuclei per fiber type II	2.3 ± 0.4	2.6 ± 0.4	2.8 ± 0.4	2.6 ± 0.4	2.1 ± 0.4	2.7 ± 0.4

Representative images of A: Fiber types I (green) and II (black), B: Cell nuclei (blue), and C: Capillaries (red). CD, capillary density; C/F-ratio, capillary-to-fiber ratio; CSA, cross-sectional area; CAF, capillaries around fibers; and CAFA, capillaries around fibers per cross-sectional area.

* Indicates *post hoc* difference from Pre (*p* < 0.05).

# Indicates main effect of group.

§ Indicates an interaction (time × group, *p* < 0.05).

## Discussion

This study compared 12 weeks of training load-matched BP, using concentrated HIT-, MIT-, and LIT-weeks, with a TP using a mix of HIT-, MIT-, and LIT-sessions and a cyclic progressive increase in training load in trained cyclists. TP and BP did not lead to different improvements in TT performances, performance-related measures, muscle protein content, or enzyme activity after 4 and 12 weeks of supervised endurance training. However, RBCV, was increased by 10% more in BP compared to TP after 12 weeks, while C/F-ratio increased 19% more in TP from Pre to Post4 but not Post12, and CAF type I increased 20% more from Pre to Post12 compared to BP. Otherwise, no differences were observed in responses to training between TP and BP. Overall, substantial improvements in performance (5 and 40-min TT power output) and performance-related measures (W_max_ and W_4mmol_), muscular protein content of HAD and COXIV, and GE in the fatigued state were evident in both BP and TP.

In the current study, no clear differences in performance or performance-related measures between the two periodization models were observed. These findings contrast the findings of a recent meta-analysis by Molmen et al. (2019), indicating a small benefit of using BP. However, the findings of the different studies included in that meta-analysis ranged from no performance-benefits of BP over TP (Breil et al., 2010; Garcia-Pallares et al., 2010; McGawley et al., 2017), to moderate, positive effects on performance and performance-related measures (e.g., VO_2peak_, W_max_, and W_4mmol_) in both trained, and highly trained athletes (Breil et al., 2010; Ronnestad et al., 2012, 2014, 2016; Clark et al., 2014; Costa et al., 2017). Differences across studies might relate to differences in training design, including duration, load administration, performance level, and distribution of training at different intensities. The present study is characterized by the combination of a long duration (12 weeks), well-controlled, supervised sessions with high adherence (92%), and a “best practice” TP with a cyclic, progressive increase in training load. Previous studies have not used such a gradual increase in numbers of MIT and HIT intervals from each week when applying a TP approach. This cyclic increase in training load could, hence, be speculated to prevent staleness in training stimuli as previously suggested (Goutianos, 2016), leading to similar improvements in performance and performance-related outcomes as BP after both shorter (4 weeks) and longer (12 weeks) training periods. Additionally, MIT exercise has rarely been included in studies comparing BP and TP and is thus a distinctive quality of our study. We found substantial improvements of several endurance performance measures such as W_max_ (6%), W_4mmol_ (~10%), and fractional utilization of VO_2_ at W_4mmol_ (~5%) in both TP and BP when including MIT, suggesting an applicability of MIT in both blocked-, and traditionally periodized programs. In support of this, Garcia-Pallares et al. (2010) included MIT and HIT intensity ranges in their comparison of BP and TP and found ~10% improvements in VO_2_ at the second ventilatory threshold. However, the use of both different durations of the MIT periods and intensity distribution during the BP and TP (Garcia-Pallares et al., 2010), makes comparisons with the present study difficult.

Additionally, the performance level and training history of our participants may have influenced our findings. The participants were generally “trained” but with rather diverging performance levels (level 2–5), making the potential for improvement highly individual. Accordingly, both groups achieved substantial improvements in performance outcomes and muscular adaptations. BP and TP groups were pair-matched based on the mean power output of a 40-min TT and total training load during the intervention. However, the individual, relative change in training load compared to habitual training load differed greatly and could be a reason for the substantial and similar improvements seen. The summary by Molmen et al. (2019) indicates that BP only has a small benefit over TP in athletes with the weighted mean VO_2peak_ of 60 ml·kg^−1^·min^−1^ (Molmen et al., 2019). It could be argued that the large and diverging improvements found in our participants would mask potential differences between BP and TP. However, the performance level of participants was similar in Molmen et al. (2019); hence, the lack of difference between BP and TP cannot fully be explained by the performance level of our participants.

Still, a previous study in a homogenous group of well-trained cyclists revealed moderate beneficial effects of BP over TP (Ronnestad et al., 2014), which could underline the need for a more concentrated training focus for athletes closer to their genetic maximum (Psilander et al., 2010) than the present participants. Another potential factor might be that our blocks included only four sessions of MIT or HIT each week, which is less than previous blocks used in studies by Ronnestad et al. (2012, 2014, 2016), where they included five sessions of HIT each week and found superior effects of BP over TP. However, the lack of a difference between BP and TP could be the result of a more successful administration of a “best practice” cyclic training load increase in TP than previous studies, leading to similar performance adaptations as BP.

The improvements of GE and performance in a fatigued state provide some additional insights. GE in the semi-fatigued state, but not in the fresh state, was improved in both BP and TP by the 12-week intervention; hence, a smaller decrease in GE from the fresh to the semi-fatigued state was observed in both groups. Previous studies in trained and competitive cyclists have reported, moderate effects in exercise economy for BP over TP, although non-significant (Clark et al., 2014; Ronnestad et al., 2014). Contrasting this, McGawley et al. (2017) showed improvement in skiing economy for TP only, although not different from BP. The 5-min time trial, which was started in a semi-fatigued state at the end of test day 1 also improved in both BP and TB. The two outcomes of improved GE and 5-min TT may have symbiotic effects because an improved GE in the fatigued state, given our current protocol, would save energy for utilization in the 5-min TT. Hence, our data support that consistent endurance training improves GE and performance in the fatiguing state in already trained athletes, which arguably is of importance for race-specific endurance performance. However, based on our findings and the cited literature, this improvement does not seem to be differently affected by the periodization model.

Interestingly, RBCV was increased in BP compared to TP, with Hb-mass showing similar numerical advantage. Although not statistically different, the numerical advantage of a 5% increase in BP (vs. −1 in TP) could be physiologically relevant. Ronnestad et al. (2012) found similar alterations in Hb-mass, ~5%, as in the present study after 12 weeks of BP, while no changes were reported for TP. However, the lack of statistical power might have influenced the present and previous conclusions as hematological measures were only performed on a subset of the participants (*n* = 14) in the present study.

Several skeletal muscle measures were altered with TP and BP, supporting the sound improvements in TT-performances herein. Muscle protein content of COXIV increased by 88 and 79% in TP and BP from Pre to Post12, while the content of HAD only increased in TP by 55%. Previously, only McGawley et al. (2017) have reported muscular adaptations to TP and BP and found a ~7% decrease in HAD-activity in BP, while CS and PFK activities, in line with our findings, were unaltered (McGawley et al., 2017). McGawley et al. (2017) did not find changes in capillary measures such as CD, which could relate to the short duration of training (3 weeks) and high performance level of the participants. Capillary adaptations are reported after 4 weeks of endurance training in previously sedentary subjects (Hellsten and Nyberg, 2015). Similarly, after 4 weeks we found a 27% increase in C/F-ratio in TP, and a 36% increase after 12 weeks. Also, CAF type I was increased by 17% in TP, whereas in BP, CD increased by 28% from Pre to Post12, underlining the sound peripheral adaptations of both periodization models.

Interestingly, C/F-ratio increased 19% more in TP from Pre to Post4, but this difference was not seen after 12 weeks. Also, CAF type I increased 20% more from Pre to Post12 compared to BP. The present diverging findings in capillarization might, however, be limited by the borderline low number of fibers included in the analysis (151 ± 105 type I fibers, and 79 ± 60 type II fibers), since an average of at least 50 fibers of each type ought to be included to acquire reliable measures (McCall et al., 1998). However, our findings support substantial improvements in muscle capillarization with both TP and BP. Although different alterations are observed between periodization models, these did not lead to different improvements of TT-performances in TP and BP.

## Practical Application

Our data does not support the superiority of one periodization model over the other when LIT, MIT, and HIT are blocked vs. when a “best practice” cyclic progressive increase in training load is applied in TP over a 12-week training period in trained cyclists. These findings are in line with a case study of the world’s best XC-skier who succeeded with both TP and BP during her career (Solli et al., 2019), supporting the applicability of both periodization models. We did not find differences in performance after 4 weeks of TP and BP; however, equivocal findings exist where BP has shown larger improvements than TP in performance measurements (Breil et al., 2010; Ronnestad et al., 2012, 2014, 2016; Clark et al., 2014; Molmen et al., 2019). Using BP as an individualized approach for coaches could be suggested when seeking to alternate training stimuli in athletes. The application of a short BP training period (e.g., a 5–7 day micro cycle) can provide a substantial overload stimulus in a targeted ability. This is not an argument for one periodization model over the other, but a proposal for BP as a tool, which can be applied to the training of athletes in specific situations.

## Conclusion

In conclusion, our data does not support the hypothesis that BP is superior to a “best practice”-TP using a cyclic, progressively increasing training load in improving the endurance performance of trained cyclists. Although hematological and muscle capillary adaptations differed, 12 weeks of training led to similar improvements in performance (5 and 40-min TT power output) and performance-related measures (W_max_ and W_4mmol_), muscular protein content of HAD and COXIV, and GE in the fatigued state in both BP and TP.

## Data Availability Statement

The raw data supporting the conclusions of this article will be made available by the authors, without undue reservation.

## Ethics Statement

The studies involving human participants were reviewed and approved by Local ethical committee at Lillehammer University College. The patients/participants provided their written informed consent to participate in this study.

## Author Contributions

NA, SE, ØS, BR, and KS contributed to conception and design of the study. NA, HE, MW, HH, SI, and KS executed the study, collected, and analyzed the data. NA and KS performed the statistical analysis and wrote the first draft of the manuscript. All authors contributed to manuscript revision, read, and approved the submitted version.

## Conflict of Interest

The authors declare that the research was conducted in the absence of any commercial or financial relationships that could be construed as a potential conflict of interest.

## Publisher’s Note

All claims expressed in this article are solely those of the authors and do not necessarily represent those of their affiliated organizations, or those of the publisher, the editors and the reviewers. Any product that may be evaluated in this article, or claim that may be made by its manufacturer, is not guaranteed or endorsed by the publisher.
